# Quantitative Analysis of Peanut Skin Adulterants by Fourier Transform Near-Infrared Spectroscopy Combined with Chemometrics

**DOI:** 10.3390/foods14030466

**Published:** 2025-02-01

**Authors:** Wangfei Luo, Jihong Deng, Chenxi Li, Hui Jiang

**Affiliations:** School of Electrical and Information Engineering, Jiangsu University, Zhenjiang 212013, China; lwf20041225@126.com (W.L.); 2112307126@stmail.ujs.edu.cn (J.D.); 2212307081@ujs.edu.cn (C.L.)

**Keywords:** peanut skin, adulteration, Fourier transform near-infrared spectroscopy

## Abstract

Peanut skin is a potential medicinal material. The adulteration of peanut skin samples with starchy substances severely affects their medicinal value. This study aimed to quantitatively analyze the adulterants present in peanut skin using Fourier transform near-infrared (FT-NIR) spectroscopy. Two adulterants, sweet potato starch and corn starch, were included in this study. First, spectral information of the adulterated samples was collected for characterization. Then, the applicability of different preprocessing methods and techniques to the obtained spectral data was compared. Subsequently, the Competitive Adaptive Reweighted Sampling (CARS) algorithm was used to extract effective variables from the preprocessed spectral data, and Partial Least Squares Regression (PLSR), a Support Vector Machine (SVM), and a Black Kite Algorithm-Support Vector Machine (BKA-SVM) were employed to predict the adulterant content in the samples, as well as the overall adulteration level. The results showed that the BKA-SVM model performed excellently in predicting the content of sweet potato starch, corn starch, and overall adulterants, with determination coefficients ($R_{P}^{2}$) of 0.9833, 0.9893, and 0.9987, respectively. The experimental results indicate that FT-NIR spectroscopy combined with advanced machine learning techniques can effectively and accurately detect adulterants in peanut skin, providing a reliable technological support for food safety detection.

## 1. Introduction

Peanut skin is the seed coat of the peanut fruit and is rich in natural antioxidants such as polyphenols, flavonoids, and sterols [1]. These components have significant antioxidant activity, which can effectively neutralize free radicals in the body, reduce oxidative damage, and play an important role in delaying aging and combating age-related degeneration [2]. In traditional medicine, peanut skin is believed to have effects such as clearing heat and detoxifying, relieving cough and phlegm, lowering blood pressure, and reducing blood lipids [3]. It is commonly used to treat various diseases caused by damp heat, especially to alleviate symptoms such as gastrointestinal discomfort and respiratory issues [4]. Additionally, the polyphenolic compounds abundant in peanut skin have potential roles in inhibiting inflammation, fighting tumors, and combating bacteria, as well as improving immune function and regulating the body’s immune response [5].

However, some unscrupulous merchants, in pursuit of higher profits, adulterate peanut skin powder with cheap starchy substances to reduce costs and increase product weight. This practice not only severely affects the purity and nutritional value of peanut skin powder, but also poses potential health risks to consumers who may inadvertently ingest unknown substances. While these adulterants are not toxic in themselves, they lack the bioactive compounds unique to peanut skin. As a result, their inclusion dilutes the effective components of peanut skin, reducing its antioxidant, anti-inflammatory, and other health benefits. Additionally, such adulteration may lead to allergic reactions, indigestion, and other health issues, potentially compromising food safety. To protect consumers’ health and rights, it is essential to establish effective detection methods, strengthen monitoring of such products, and ensure that peanut skin powder products in the market meet standards and are genuine.

Currently, various detection techniques have been applied to address the issue of food adulteration. High-Performance Liquid Chromatography (HPLC) [6] and Gas Chromatography (GC) [7] can separate the different components of a sample and perform quantitative analysis, offering high sensitivity and accuracy. Sun et al. used HPLC-DAD to detect adulteration in cinnamon samples [8]. But due to the need for meticulous sample preparation and chromatographic analysis, this method is time-consuming and not suitable for rapid screening. Shi et al. used HPLC-DAD to analyze adulterants in different grades of camellia oil [9], distinguishing different grades of camellia oil by analyzing their volatile compounds. However, this method requires a large sample size and is complex, making it suitable only for laboratory environments. Additionally, molecular biology detection methods based on DNA barcoding have been developed [10]. Uncu et al. used HPLC-DAD to analyze adulterants in plant oils in dairy products [11], identifying adulterants by amplifying plant-specific genetic regions. However, this process is complex and time-consuming and, thus, cannot meet the need for real-time detection. Therefore, there is an urgent need for a detection method that is accurate, efficient, and quick to operate, in order to comprehensively identify adulterants in food for food safety testing.

Fourier Transform Near-Infrared (FT-NIR) spectroscopy is an efficient, non-destructive, and environmentally friendly detection method that has gained widespread application in the food safety sector in recent years [12]. Compared to traditional chemical analysis methods, FT-NIR offers higher resolution and a broader spectral range, enabling the rapid and accurate identification of chemical features in peanut skin, such as the content and distribution of components like proteins and polysaccharides [13]. Through the Fourier transformation process, FT-NIR converts spectral signals into frequency domain information, making data processing more efficient. This technique is not only easy to operate with simple equipment, but also does not rely on chemical reagents, meeting the requirements of green testing [14]. In fact, several studies have demonstrated the application of FT-NIR in food testing. For instance, Deng et al. [15] used FT-NIR spectroscopy combined with linear and nonlinear models to accurately detect mineral oil contamination in corn oil, and Meng et al. [16] employed FT-IR spectroscopy combined with chemometrics to quickly detect adulteration in olive oil and soybean oil. These results indicate that FT-NIR, when combined with chemometrics, can establish precise quantitative analysis models. However, most current research on adulteration remains focused on analyzing single adulterants in samples, which often does not reflect real-world scenarios [17]. Therefore, comprehensive studies on multiple adulterants in samples are particularly urgent [18].

In this context, the aim of this study is to propose a non-destructive and efficient method for rapidly analyzing the content of starchy adulterants in peanut skin samples. The specific objectives are as follows: (1) Use FT-NIR to acquire spectral data of peanut skin samples with different adulteration ratios and preprocess the data. (2) Optimize the feature wavelengths of the preprocessed data and introduce the CARS algorithm. (3) Based on the optimized feature wavelengths, apply PLSR, SVM, and BKA-SVM models for quantitative analysis and comparison of the adulterant content in peanut skin.

## 2. Materials and Methods

### 2.1. Sample Preparation

The goal of this study is to explore the potential of FT-NIR in the quantitative analysis of starchy adulterants in peanut skin. Two adulterants, sweet potato starch and corn starch, were prepared and sourced through online channels. Additionally, 5 pounds of peanut skin was purchased from a supermarket.

In the sample preparation stage, the peanut skin was first placed in a multifunctional pulverizer (Deqing Baijie Electric Co., Ltd., Huzhou, China) in a dry, cool environment, with the grinding time set to 2 min to ensure that it was finely powdered. The powdered peanut skin was then sealed in air-extracted packaging bags for storage. Next, a sampling spoon was used to take sweet potato starch and corn starch in different ratios of 1:1, 2:3, 3:2, 2:1, and 1:3, and place it on weighing paper. The exact weight of the samples was measured using a pre-calibrated electronic balance. Afterward, the powdered peanut skin was added to the adulterants to simulate different levels of adulteration and the mixtures were sealed in air-extracted bags for storage. Finally, the samples were taken from the bags and placed in test tubes, which were then put on a shaker (Eppendorf AG, Hamburg, Germany) for 2 min to ensure thorough mixing. This resulted in 15 different concentration gradients: 40, 36, 30, 24, 20, 16, 12, 8, 6, 4, 3, 2, 1, 0.5, and 0%. The specific content of each adulterant at each concentration is shown in Table S1. A total of 15 independent experimental samples were prepared for each concentration. Therefore, a total of 225 peanut skin samples with varying adulteration levels were prepared for the experiment. Additionally, 3 additional control samples (100% peanut skin, 100% sweet potato starch, and 100% corn starch) were prepared for the experiment.

### 2.2. Spectral Acquisition

In this experiment, Fourier Transform Near-Infrared (FT-NIR) spectroscopy (Antaris™II, Thermoelectric) was used to collect spectral data from peanut skin samples. Prior to each data collection session, the spectrometer was preheated for 30 min to reach a stable state. A 5 g sample of peanut skin with varying levels of adulteration was placed in a quartz glass container, and spectra were recorded using the diffuse reflectance method for spectral data collection. Before operation, the instrument was precisely calibrated with a spectral resolution set to 8 cm^−1^, a number of scans per sample equaling 64, an infrared light scanning area of 176.71 mm^2^, and a wavelength range covering 10,000 to 4000 cm^−1^. Each sample was scanned three times during the measurement process. After each measurement, the powder was flipped and compacted before the next scan. Finally, the average of the three results was taken as the initial spectrum for the sample. The temperature of the experimental environment was maintained at around 25 °C to ensure stable measurement conditions.

### 2.3. Chemometric Analysis

This study uses FT-NIR spectroscopy for the quantitative analysis of the content of sweet potato starch, corn starch, and overall adulterants in peanut skin samples. The same sample set was used throughout the study. The sweet potato starch contained in the sample set is denoted as Adulterant-A, the corn starch is denoted as Adulterant-B, and the total adulterant content (combination of both starches) is denoted as Adulterant-C.

#### 2.3.1. Spectral Data Processing

Spectral data are often influenced by background noise, baseline drift, and scattering effects, which can reduce the accuracy of data processing and affect the modeling results [19]. Therefore, before analyzing the FT-NIR spectral data of peanut skin adulteration samples, appropriate data preprocessing is essential [20]. To this end, various preprocessing methods were compared in order to evaluate their effect on the spectral data.

To minimize the impact of scattering effects, the Multivariate Scatter Correction (MSC) [21] and Standard Normal Variate (SNV) [22] methods were applied. MSC corrects the linear scattering effects caused by uneven particle distribution and size differences in the samples, improving the consistency of the spectral data. Similarly, SNV normalizes the spectral data by removing baseline drift and scaling effects, ensuring that the spectra accurately reflect the chemical composition of each sample. Additionally, to reduce noise interference, the Savitzky–Golay (SG) smoothing method was used [23]. This method smooths random noise in the spectral data by performing polynomial fitting within a defined window size while preserving important spectral features. In this study, the SG polynomial order was set to 2, and the window size was set to 11.

#### 2.3.2. Feature Selection

Competitive Adaptive Reweighted Sampling (CARS) is a feature selection method based on the Monte Carlo sampling principle, mimicking Darwin’s “survival of the fittest” concept [24]. The CARS algorithm selects important wavelengths from the original spectral data using an Adaptive Reweighted Sampling (ARS) strategy. In each iteration, CARS assigns weights to wavelengths, retaining those with larger weights and discarding those with smaller weights [25]. By repeatedly performing this process, the algorithm ultimately selects the most predictive wavelengths. Initially, each wavelength is assigned a weight, and these weights are adjusted through iterations, gradually eliminating wavelengths that contribute less to the model. After each update, the subset of wavelengths is evaluated using the Root Mean Squared Error of Cross-Validation (RMSECV). The final selected wavelengths are those that significantly improve the predictive performance and reduce the error. This method not only reduces the dimensionality of spectral data, but also effectively avoids overfitting, making it particularly suitable for feature selection and dimensionality reduction in high-dimensional data.

#### 2.3.3. Quantitative Models

Partial Least Squares Regression (PLSR) is a statistical method used to establish linear relationships between independent and dependent variables, primarily employed for dimensionality reduction in high-dimensional data [26]. PLSR works by identifying the combinations of independent variables that can most effectively explain variations in the dependent variable, thus overcoming issues of multicollinearity between predictors. By analyzing the covariance structure between the independent and dependent variables, it selects the most predictive linear components, effectively reducing redundancy and improving model accuracy [27]. The advantage of PLSR lies in its ability to handle multicollinearity issues efficiently while simultaneously reducing dimensionality, which improves the stability and interpretability of the model. However, PLSR may have limited effectiveness when dealing with complex nonlinear relationships, as it primarily relies on linear assumptions. Additionally, PLSR does not inherently provide a variable selection feature, so in high-dimensional data, it may retain irrelevant or redundant features. In this study, the number of latent variables for PLSR was selected through five-fold cross-validation, with the range of latent variables set from 1 to 20 to optimize the model’s predictive performance.

The Support Vector Machine (SVM) method is a widely used machine learning method in various fields [28]. The core idea is to minimize structural risk in order to improve the model’s generalization ability, thereby reducing empirical risk and confidence intervals. An SVM effectively captures statistical patterns in data and can handle linearly non-separable problems [29]. Typically, it maps data to a higher-dimensional space using a kernel function. The Radial Basis Function (RBF) kernel is a commonly used function that plays a crucial role in the application of SVMs. To optimize model performance, a combination of five-fold cross-validation and grid search is often employed to adjust the penalty coefficient c and the kernel function parameter g [30].

However, grid search has certain limitations when searching for the optimal parameters. It has a large computational overhead, and its efficiency is relatively low, often getting stuck in local optima. To overcome these limitations, the Black Kite Algorithm-Support Vector Machine (BKA-SVM) method was introduced. The BKA algorithm is a metaheuristic optimization method based on the behavior simulation of the Black Kite Algorithm (BKA). It simulates the foraging, gliding, and attacking behaviors of the black kite in nature, demonstrating strong global search capability and local exploitation ability. Compared to traditional optimization algorithms, BKA has the advantages of simplicity, fast convergence speed, and strong global optimization ability, making it particularly suitable for solving high-dimensional, nonlinear, and multi-objective optimization problems. BKA-SVM combines the BKA algorithm with SVM, using BKA to optimize key parameters such as cc and gg in SVM, thus achieving better fitting of complex nonlinear relationships.

### 2.4. Model Evaluation

Model performance is typically assessed by analyzing the predictive ability, with common evaluation metrics including the coefficient of determination (R^2^) and the Root Mean Square Error (RMSE). Generally, higher R^2^ values and lower RMSE values indicate better model performance. The Root Mean Square Error of Calibration (RMSEC) is used to evaluate the model’s fitting ability in relation to the training data. Its value reflects the error between the model’s predictions and the known data during the calibration process. A lower RMSEC indicates better learning with the training set but does not guarantee the model’s performance when using new data. The Root Mean Square Error of Prediction (RMSEP) is used to measure the model’s generalization ability, i.e., its prediction performance with the test data set. By calculating the deviation between the predicted and actual values, RMSEP reflects the model’s ability to adapt to new data. Smaller RMSEP values indicate higher prediction accuracy for unseen data, demonstrating the model’s reliability in practical applications [31].

## 3. Results and Discussion

### 3.1. Dividing the Samples into Prediction and Calibration Sets

To avoid random errors and aid in building a robust model, this study adopts the Kennard–Stone (KS) algorithm for sample splitting. The KS algorithm selects samples based on their distribution uniformity, prioritizing those that are evenly distributed in the chemical space to form the training set, thereby ensuring the representativeness of the training set [32]. Additionally, the KS algorithm effectively avoids issues related to uneven sample distribution or missing chemical information, allowing the prediction set to comprehensively evaluate the model’s generalization ability. In this study, the KS algorithm is used to divide the peanut skin adulteration samples, with 158 samples allocated to the training set and the remaining 67 samples assigned to the prediction set.

**Figure 1:**
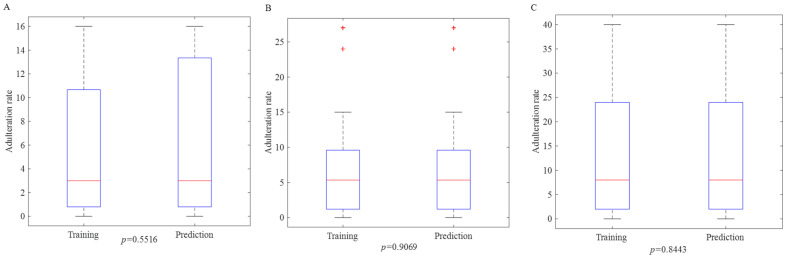
A comparison of the boxplots of the distribution of adulteration rates between the training and prediction sets. (**A**): Adulteration-A; (**B**): Adulteration-B; (**C**): Adulteration-C.

A boxplot is used to assess the division of the dataset by visually displaying the distribution, median, data range, and outliers of both the training and prediction sets. This allows for an intuitive evaluation of the consistency and balance between the two groups, helping to determine whether the split is reasonable. As shown in Figure 1, the significance test results (*p*-values) for Adulterant-A, Adulterant-B, and Adulterant-C are 0.5516, 0.9069, and 0.8433, respectively, all of which are greater than 0.05 [33]. This indicates that the mean differences between the training and prediction sets for each adulterant are not statistically significant, suggesting that the split is effective. However, it is worth noting that in the Adulterant-B test, both the training and prediction sets exhibit outliers at higher adulteration levels. This is because the Adulterant-B ratio follows a long-tailed distribution, with most samples having adulteration levels below 10%, but a small number of samples with higher levels (24% and 27%) appear in the tail. Since the outliers are similarly distributed in both the training and prediction sets, they do not negatively affect the model’s training or prediction. In fact, these outliers provide an opportunity for the model to learn from extreme cases, which can enhance the model’s ability to predict boundary samples.

### 3.2. Spectral Characteristics

**Figure 2:**
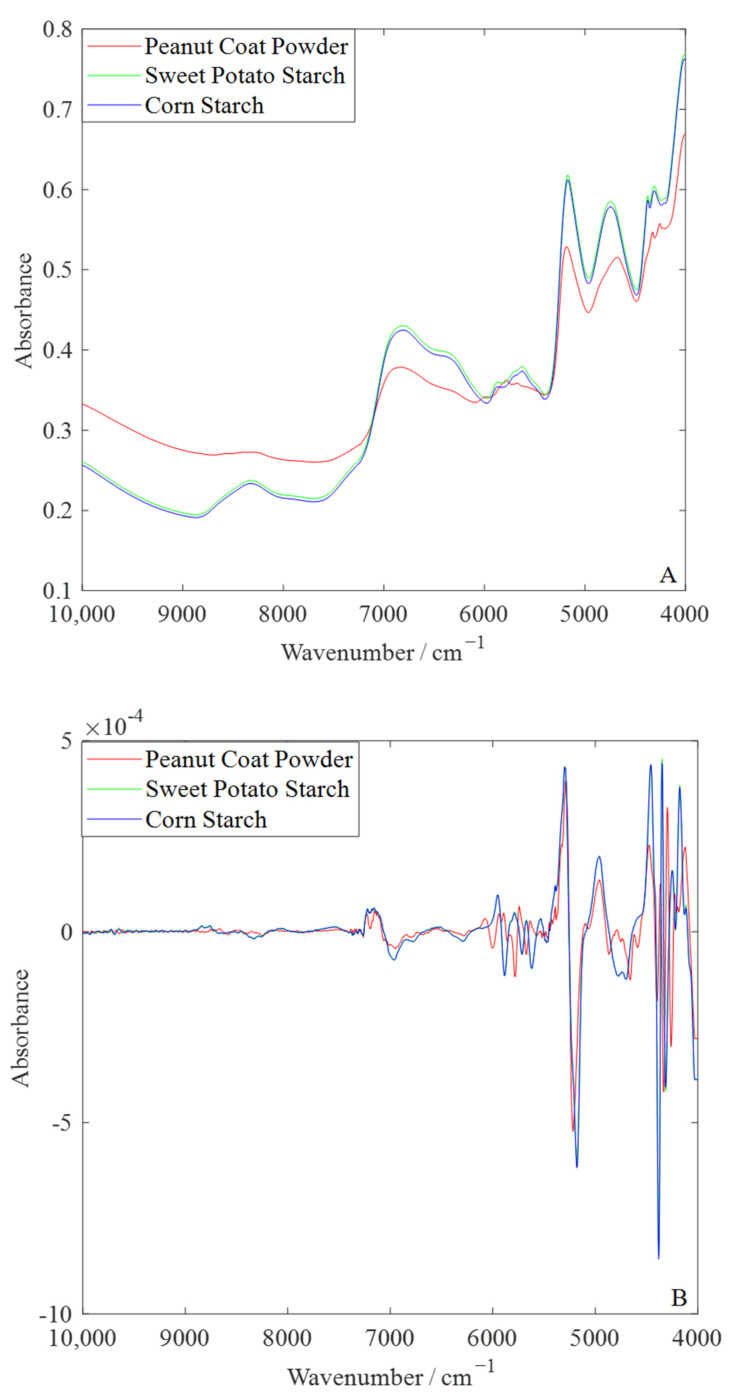
The FT-NIR spectra of the pure samples. (**A**): Original FT-NIR spectra; (**B**): second derivative spectra.

The spectral acquisition of the pure samples is shown in Figure 2A. In the FT-NIR spectrum, peanut skin powder exhibits several prominent absorption peaks, reflecting its complex chemical composition and characteristic molecular vibrations. At around 4700 cm^−1^, a significant absorption peak is observed, which is associated with the absorption band of cis double-bond fatty acids [34]. This peak shape serves as a unique marker for peanut skin powder and distinguishes it from the common C-H vibration absorption observed in sweet potato starch and corn starch. Within the range of 5000–6000 cm^−1^, two strong absorption peaks of peanut skin powder are related to overtones of C-H stretching vibrations, indicating its more complex molecular vibration patterns [35]. In contrast, the absorption peaks of sweet potato starch and corn starch in this range are more symmetrical, suggesting simpler molecular structures predominantly governed by OH and C-H group vibrations. In the 8200–8400 cm^−1^ region, the absorption peaks of peanut skin powder are relatively smooth, possibly due to its more uniform molecular structure and the presence of long-chain unsaturated fatty acids. On the other hand, starch samples exhibit weaker absorption peak intensities in this region, further highlighting the compositional differences.

In order to further analyze the spectral differences between peanut coat powder, sweet potato starch, and corn starch, the FT-NIR spectra of the pure samples were subjected to second derivative processing. As shown in Figure 2B, the characteristic peaks of peanut coat powder are sharper and more pronounced, especially in the 4700 cm^−1^ and 5000–6000 cm^−1^ regions. The absorption peaks in these regions are significantly enhanced in the second derivative spectra, making the differences between peanut coat powder and sweet potato starch and corn starch more pronounced. At around 4700 cm^−1^, peanut coat powder displays a strong absorption peak, further highlighting its characteristic cis double-bond fatty acids. Meanwhile, the absorption peaks in the 5000–6000 cm^−1^ region are unique to peanut coat powder, reflecting its more complex molecular vibration patterns, whereas sweet potato starch and corn starch have more symmetrical absorption peaks in these regions.

### 3.3. Analysis of Spectral Preprocessing Results

**Figure 3:**
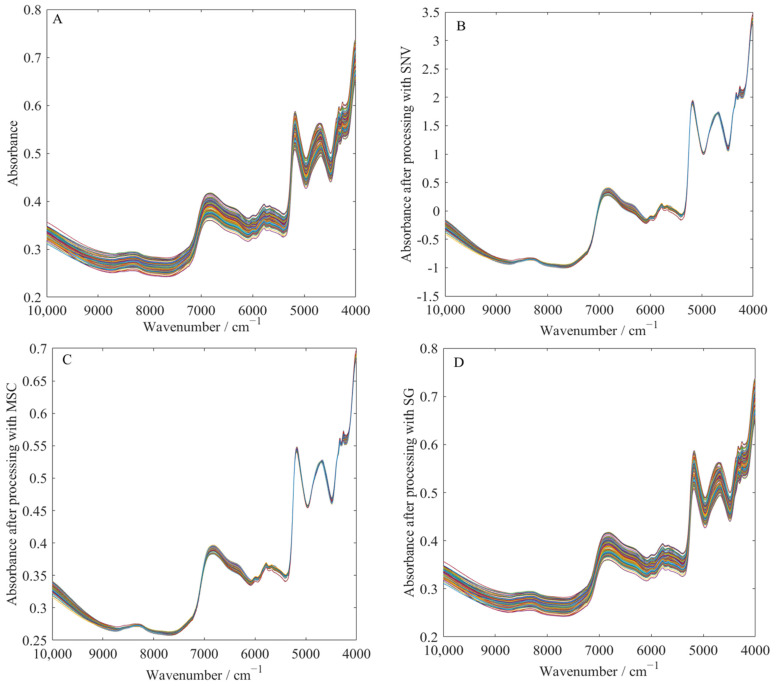
The results of the different spectra preprocessing methods. (**A**): Raw; (**B**): SNV; (**C**): MSC; (**D**): SG.

As shown in Table 1 and Figure 3, the spectra processed by different preprocessing methods achieve lower RMSEP and higher $R_{P}^{2}$ compared to the original spectra, significantly improving the prediction performance. Furthermore, SG smoothing treatment consistently outperforms the other methods in the detection of all adulterants, always achieving the lowest RMSEP and the highest $R_{P}^{2}$.

This improvement is due to the significant enhancement of spectral quality after preprocessing. SNV treatment (Figure 3B) corrects for scattering effects, aligning the spectral curves of different samples by standardizing the baseline. MSC treatment (Figure 3C) also corrects for scattering effects while reducing baseline drift, maintaining spectral smoothness, and preventing noise amplification, thus providing a more stable foundation for further data analysis. SG smoothing treatment (Figure 3D) focuses on reducing high-frequency noise interference, making the spectral curve smoother while preserving important absorption peak features, which is particularly helpful for identifying subtle changes in the spectrum.

### 3.4. Prediction Results for Different Adulterations

**Figure 4:**
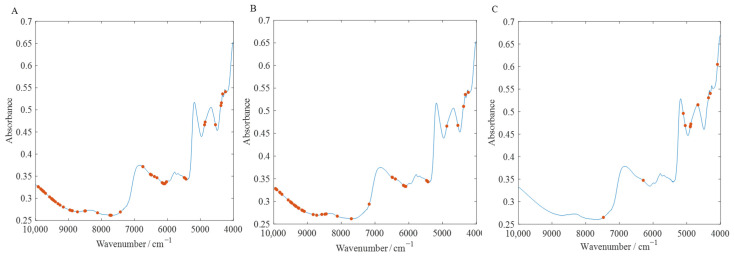
The distribution of the feature variables of the full spectrum based on the CARS algorithm. (**A**): Adulteration-A; (**B**): Adulteration-B; (**C**): Adulteration-C.

**Figure 5:**
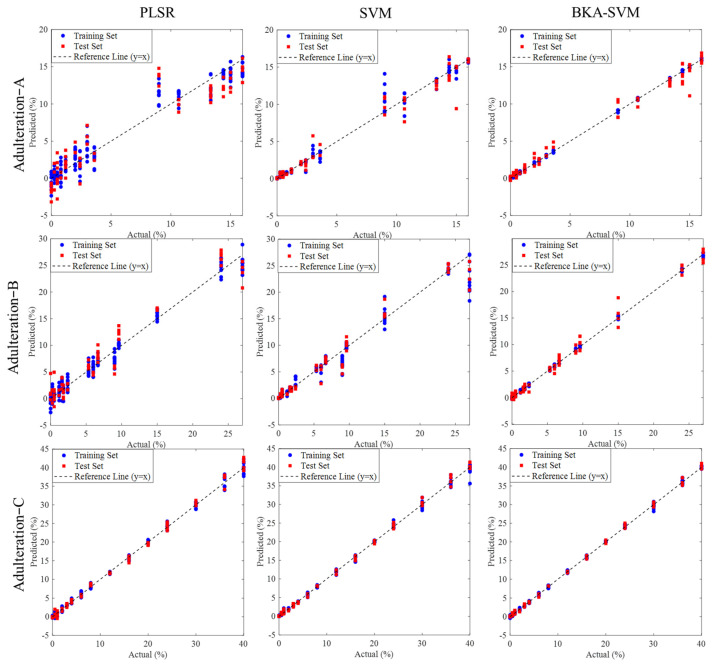
Scatter plots of the predictions by different models.

As shown in Figure 4A, Adulteration-A, under CARS feature selection, selected 60 variables, which account for 3.8% of the total variables. Based on this, different models were used for prediction. Table 2 presents the results of the various models. When the optimal latent variables for PLSR were 11, the RMSECV was minimized, with $R_{C}^{2}$ = 0.9365, RMSEC = 1.6145%, $R_{P}^{2}$ = 0.8911, RMSEP = 2.0470%. For SVM, when c = 2.8284, g = 0.0221, the RMSECV was lowest, with $R_{C}^{2}$ = 0.9853, RMSEC = 0.7051%, $R_{P}^{2}$ = 0.9713, RMSEP = 1.0518%. However, for the BKA-SVM model, when the RMSECV was minimized, the best parameters were c = 431.3487, g = 0.0405, with $R_{C}^{2}$ = 0.9930, RMSEC = 0.1520%, $R_{P}^{2}$ = 0.9833, RMSEP = 0.8026%. Combining the analysis above and the scatter plots shown in Figure 5—which illustrate the predicted vs. actual values for Adulteration-A across the models—makes it clear that the BKA-SVM model produced the best performance.

As shown in Figure 4B, Adulteration-B, under CARS feature selection, selected 42 variables, which account for 2.7% of the total variables. Based on this, different models were used for prediction. The results of the various models can be observed in Table 2 and Figure 4B. When the optimal latent variables for PLSR were 10, the RMSECV was minimized, with $R_{C}^{2}$ = 0.9815, RMSEC = 1.3982%, $R_{P}^{2}$ = 0.9375, RMSEP = 2.0544%. For the SVM model, when the parameters c = 22.6274, g = 0.0028, the RMSECV was lowest, with $R_{C}^{2}$ = 0.9658, RMSEC = 1.5203%, $R_{P}^{2}$ = 0.9579, RMSEP = 1.6909%. In contrast, the BKA-SVM model demonstrated a superior prediction performance. When the RMSECV was minimized, the corresponding optimal parameters were c = 1020.2249, g = 0.0141, yielding $R_{C}^{2}$ = 0.9990, RMSEC = 0.2624%, $R_{P}^{2}$ = 0.9893, RMSEP = 0.8494%. Based on the above analysis and the scatter plots of the predicted values versus actual values for the Adulteration-B prediction set shown in Figure 5, it is evident that the BKA-SVM model performs the best. Its fit and precision are significantly superior to those of the other models.

Similarly, for Adulteration-C, the 12 variables selected using the CARS feature selection method can be seen in Figure 4C. Table 2 shows the results of the different models used for the prediction of Adulteration-C. When the optimal number of latent variables for PLSR is nine, the RMSECV is minimized, with $R_{C}^{2}$ = 0.9971, RMSEC = 0.7033%, $R_{P}^{2}$ = 0.9960, RMSEP = 0.8014%. For SVM, when c = 64, g = 0.0009, the RMSECV is minimized, with $R_{C}^{2}$ = 0.9978, RMSEC = 0.6180%, $R_{P}^{2}$ = 0.9977, RMSEP = 0.6225%, and RMSEP = 0.6225%. Based on the scatter plot of the predicted vs. actual values for Adulteration-C shown in Figure 5, it can be seen that the BKA-SVM model outperforms the other models in terms of fit accuracy and prediction performance. In contrast, the BKA-SVM model not only achieves higher $R_{C}^{2}$ = 0.9978, $R_{P}^{2}$ = 0.9977, but also significantly reduces the RMSEC and RMSEP values to 0.4003% and 0.4801%, respectively. This demonstrates its stronger fitting capability and higher prediction accuracy. Therefore, the BKA-SVM model exhibits the best overall performance in handling the Adulteration-C prediction task and provides the most reliable prediction results.

### 3.5. Discussion

#### 3.5.1. Discussion of Selected Variables

Figure 4 displays the results of CARS feature selection using the FT-NIR spectral data of the peanut skin adulteration samples. For Adulterant-A, the feature variables are primarily concentrated in the wavenumber ranges of 4000–5000 cm^−1^ and 6000–7000 cm^−1^. The absorption peaks in these bands are typically associated with the vibrational responses of C-H and O-H bonds. Specifically, the peak around 4200–4400 cm^−1^ may correspond to the first overtone of C-H [36], while the absorption peak near 6800 cm^−1^ could reflect the second stretching overtone of O-H bonds, suggesting that the chemical characteristics of sweet potato are mainly related to the presence of hydrocarbons and hydroxyl groups.

For Adulterant-B, the feature variables are more widely distributed and are primarily concentrated in the ranges of 4500–5500 cm^−1^ and 7500–9000 cm^−1^. The 5000–5500 cm^−1^ band generally corresponds to the second overtone of C-H combinations, while the band around 8000 cm^−1^ is associated with the stretching vibration combination band of O-H and C-H [37]. This indicates that the chemical characteristics of corn are dominated by carbon-hydrogen bonds and hydroxyl groups, which are potentially linked to the moisture characteristics in its starch composition.

In the overall adulterant analysis, the feature variables are mainly concentrated in the 4000–5000 cm^−1^ and 6000–7000 cm^−1^ ranges. The absorption peaks in these bands reflect the common features of both sweet potato and corn, particularly the hydroxyl (O-H) and carbon-hydrogen (C-H) bonds and moisture characteristics in starch. Overall, the key bands selected by CARS effectively capture the chemical characteristics of the three adulterants, with the 4000–5000 cm^−1^ and 6000–7000 cm^−1^ ranges showing consistency and stability. This demonstrates the scientific validity and reliability of FT-NIR spectroscopy in detecting peanut skin adulteration. These analytical results provide a chemical basis and technical support for efficient and accurate detection of adulterant content.

#### 3.5.2. Discussion of Different Predicting Models

For the prediction of Adulterant-A, Adulterant-B, and overall adulterant content, all models exhibited high prediction accuracy, but there were significant differences between the models. In the PLSR model, although reasonable prediction performance was achieved, the RMSEP of the test set was notably higher compared to the other two models. In fact, for Adulterant-A prediction, the RMSEP was nearly twice that of the SVM model. This is likely because the PLSR model assumes a linear relationship between the variables and is unable to capture the complex nonlinear features present in spectral data. So, the PLSR model is not suitable for detecting Adulterant-A.

In contrast, the SVM model is better suited to handling nonlinear data. By introducing a kernel function, an SVM can fit complex nonlinear relationships in a high-dimensional feature space, thereby improving prediction performance. However, SVMs are highly sensitive to the hyperparameters cc and gg, which significantly affect their performance. Improper parameter selection can lead to overfitting or underfitting, limiting the model’s predictive power.

The BKA-SVM model optimizes the parameters further than the SVM model, significantly enhancing its fitting ability. This improvement compensates for the shortcomings of the grid search in selecting the optimal c and g parameters for SVM. During the prediction of Adulterant-B and the overall adulterant content, the RMSEP values reached 0.8494% and 0.4801%, respectively, which are much lower than those of PLSR and SVM. This suggests that the BKA-SVM model is better equipped to handle the high dimensionality and complexity of spectral data, enabling it to capture the chemical information of samples more effectively.

## 4. Conclusions

This study combined FT-NIR spectroscopy and chemometrics to conduct quantitative analysis of peanut skin adulterant samples, validating the feasibility of this technique for non-destructive food safety monitoring. The study found that SG smoothing preprocessing yielded the best results for adulterant spectra. Based on this, key characteristic wavelengths were extracted using the CARS algorithm, and various predictive models including PLSR, SVM, and BKA-SVM were established. The results indicate that the BKA-SVM model performs the best in handling complex nonlinear spectral data, with $R_{P}^{2}$ values greater than 0.98 for all adulterant models. The predictions for sweet potato starch content, corn starch content, and overall adulterant content had $R_{P}^{2}$ values of 0.9833, 0.9893, and 0.9987, respectively, demonstrating superior fitting ability and prediction accuracy. These findings lay the groundwork for further applications of FT-NIR technology in food quality testing.

## Figures and Tables

**Table 1:** Statistical results of different spectral preprocessing methods.

Indicators	Methods	Parameters	Train		Test	
			$R_{C}^{2}$	RMSEC (%)	$R_{P}^{2}$	RMSEP (%)
Adulteration-A	Raw	Lvs = 12	0.9092	1.7557	0.8709	2.2281
	SNV	Lvs = 12	0.9029	1.8152	0.8790	2.1571
	MSC	Lvs = 10	0.8940	1.8969	0.8761	2.1833
	SG	Lvs = 11	0.8853	1.9727	0.8834	2.1175
Adulteration-B	Raw	Lvs = 8	0.9245	2.2643	0.9235	2.2738
	SNV	Lvs = 7	0.9353	2.0953	0.9311	2.1571
	MSC	Lvs = 11	0.9313	2.1592	0.9275	2.2135
	SG	Lvs = 10	0.9433	1.9614	0.9315	2.1510
Adulteration-C	Raw	Lvs = 7	0.9959	0.8360	0.9956	0.8866
	SNV	Lvs = 8	0.9960	0.8228	0.9957	0.8732
	MSC	Lvs = 8	0.9972	0.6905	0.9957	0.8738
	SG	Lvs = 9	0.9974	0.6648	0.9958	0.8695

**Table 2:** Results of different models for predicting adulterant content in peanut skin samples.

Indicators	Models	Parameters	Train		Test	
			$R_{C}^{2}$	RMSEC (%)	$R_{P}^{2}$	RMSEP (%)
Adulteration-A	PLSR	Lvs = 11	0.9365	1.6145	0.8911	2.0470
	SVM	c = 2.8284 g = 0.0221	0.9853	0.7051	0.9713	1.0518
	BKA-SVM	c = 431.3487 g = 0.0405	0.9930	0.1520	0.9833	0.8026
Adulteration-B	PLSR	Lvs = 10	0.9815	1.3982	0.9375	2.0544
	SVM	c = 22.6274 g = 0.0028	0.9658	1.5203	0.9579	1.6909
	BKA-SVM	c = 1020.2249 g = 0.0141	0.9990	0.2624	0.9893	0.8494
Adulteration-C	PLSR	Lvs = 9	0.9971	0.7033	0.9960	0.8014
	SVM	c = 64 g = 0.0009	0.9978	0.6180	0.9977	0.6225
	BKA-SVM	c = 1020.7405 g = 0.0018	0.9991	0.4003	0.9987	0.4801

## Data Availability

The original contributions presented in this study are included in the article and Supplementary Material. Further inquiries can be directed to the corresponding author.

## Supplementary Materials

The following supporting information can be downloaded at: https://www.mdpi.com/article/10.3390/foods14030466/s1, Table S1: Specific amount of each adulterated substance at each concentration.
