# Supplementary material for: Quantitative Analysis of Peanut Skin Adulterants by Fourier Transform Near-Infrared Spectroscopy Combined with Chemometrics
Source: Foods. 2025 Feb 1;14(3):466. doi: 10.3390/foods14030466 (PMC11817778; doi:10.3390/foods14030466)
Supplement: Supplementary file 1 [file foods-14-00466-s001.zip › foods-3438616-supplementary.pdf]

Table S1 Specific amount of each adulterated substance at each concentration.

serial number	quality ratio	sweet potato starch	corn starch	Overall adulterants
1	0:0	0%	0%	0%
2	1:1	0.25%	0.25%	0.5%
3	1:1	0.5%	0.5%	1%
4	2:3	0.8%	1.2%	2%
5	2:3	1.2%	1.8%	3%
6	3:2	2.4%	1.6%	4%
7	3:2	3.6%	2.4%	6%
8	1:3	2%	6%	8%
9	1:3	3%	9%	12%
10	2:1	10.7%	5.3%	16%
11	2:1	13.3%	6.7%	20%
12	3:2	14.4%	9.6%	24%
13	1:1	15%	15%	30%
14	1:3	9%	27%	36%
15	2:3	16%	24%	40%
